# The Sycamore Maple Bacterial Culture Collection From a TNT Polluted Site Shows Novel Plant-Growth Promoting and Explosives Degrading Bacteria

**DOI:** 10.3389/fpls.2018.01134

**Published:** 2018-08-03

**Authors:** Sofie Thijs, Wouter Sillen, Sascha Truyens, Bram Beckers, Jonathan van Hamme, Pieter van Dillewijn, Pieter Samyn, Robert Carleer, Nele Weyens, Jaco Vangronsveld

**Affiliations:** ^1^Environmental Biology, Centre for Environmental Sciences, Hasselt University, Diepenbeek, Belgium; ^2^Department of Biological Sciences, Thompson Rivers University, Kamloops, BC, Canada; ^3^Estación Experimental del Zaidín, Consejo Superior de Investigaciones Científicas, Granada, Spain; ^4^Applied and Analytical Chemistry, Centre for Environmental Sciences, Hasselt University, Diepenbeek, Belgium

**Keywords:** plant-associated bacteria, *Acer pseudoplatanus*, plant-growth-promoting-bacteria, TNT degradation, culture collections

## Abstract

Military activities have worldwide introduced toxic explosives into the environment with considerable effects on soil and plant-associated microbiota. Fortunately, these microorganisms, and their collective metabolic activities, can be harnessed for site restoration via *in situ* phytoremediation. We characterized the bacterial communities inhabiting the bulk soil and rhizosphere of sycamore maple (*Acer pseudoplatanus*) in two chronically 2,4,6-trinitrotoluene (TNT) polluted soils. Three hundred strains were isolated, purified and characterized, a majority of which showed multiple plant growth promoting (PGP) traits. Several isolates showed high nitroreductase enzyme activity and concurrent TNT-transformation. A 12-member bacterial consortium, comprising selected TNT-detoxifying and rhizobacterial strains, significantly enhanced TNT removal from soil compared to non-inoculated plants, increased root and shoot weight, and the plants were less stressed than the un-inoculated plants as estimated by the responses of antioxidative enzymes. The sycamore maple tree (SYCAM) culture collection is a significant resource of plant-associated strains with multiple PGP and catalytic properties, available for further genetic and phenotypic discovery and use in field applications.

## Introduction

Terrestrial sites polluted with explosive compounds such as 2,4,6-trinitrotoluene (TNT) are widespread and present persistent environmental problems (Nishino and Spain, 2004; Kulkarni and Chaudhari, 2007). Pollution can occur during manufacturing, transport, storage of obsolete ammunition, burning and detonation operations, especially at military installations (Rieger and Knackmuss, 1995; Singh and Mishra, 2014). The impacts of TNT pollution are exacerbated by the large amounts of water that are required for its production, which eventually end up in aqueous waste streams that may be discharged on soil or in shallow basins severely polluting soil and (ground)water (Kalafut et al., 1998; Brannon and Pennington, 2002). The TNT concentrations in polluted water and soils can reach as high as 20,000 mg TNT kg^−1^ (Rieger and Knackmuss, 1995). Because TNT is toxic, mutagenic and a potential carcinogen, there is a strong incentive for remediating these locations. The remoteness of military sites, large area, and diffuse spread of explosives pollution, directs attention toward using green and sustainable technologies such as phytoremediation in a way to reduce prohibitive high costs associated with classical dig and dump (Kilian et al., 2001; Letzel et al., 2003; Khan et al., 2013).

Plant roots are continuously exposed to diverse microbial communities and there exist strong interactions and dynamics between the host plant and its microbiome. Exudates released by plant roots can provide nutrients and energy for bacteria, while several community members in turn beneficially influence vegetation dynamics (Chaudhry et al., 2004; Mackova et al., 2009; Knief et al., 2011; Segura and Ramos, 2013). In general, there is a close relationship between the plant host and its rhizosphere microbiota, however, pollution may alter these interactions. Plants may ‘call’ on pollutant degrading bacteria while other pollutant-sensitive but plant-specific taxa may significantly decrease in abundance (Siciliano et al., 2001; Bell et al., 2014; Yergeau et al., 2014). Native plants that are spontaneously colonizing TNT polluted soils are ideal models to study in an attempt to better understand how bacterial communities respond to environmental pollution.

Owing to the presence of TNT polluted locations worldwide, several efforts have focused on the development of efficient remediation strategies (Clark and Boopathy, 2007; Mulla et al., 2013). Bioremediation has been considered advantageous because bacterial enzymatic reactions are diverse and have the potential to degrade a diverse set of organic compounds (Parke et al., 2000; Ramos et al., 2005; Rylott et al., 2011b). In general, TNT-transformation in aerobic soils is mediated by bacterial nitroreductases found amongst the genera *Achromobacter*, *Enterobacter*, *Klebsiella*, *Pseudomonas*, and others leading to the formation of amino-metabolites which are less bioavailable and less toxic than the parent compound (Boopathy et al., 1994; French et al., 1998; Labidi et al., 2001; Kim et al., 2002; Caballero et al., 2005; Gonzalez-Perez et al., 2007; Neuwoehner et al., 2007; van Dillewijn et al., 2008b; Rahal and Moussa, 2011; Thijs et al., 2014a; Iman et al., 2017). However, natural TNT degradation is extremely slow due to the chemical structure of TNT which renders it particularly resistant to oxidative attack, ring cleavage and thus mineralization (Qasim et al., 2007). In addition, TNT can have a low bio-availability as a consequence of irreversible binding with humic acids, clay and organic matter, and also harsh environmental conditions, e.g., nutrient limitations, co-pollutants and high toxicity, are not promotive of TNT degradation. As such, novel plant-growth promoting strains with TNT-degrading properties ought to be mined to improve current phytoremediation practices.

While one microorganism may play a dominant role in a particular degradation process, the contribution of associated microbial strains in a consortium may be crucial in augmenting phytoremediation effectiveness (Snellinx et al., 2003). Microbial consortia can be the solution to deal with the multi-complexity of TNT polluted soils, as the partnership might synergistically improve plant biomass, root formation, nutrient availability and toxicity reduction. Though these parameters need to be experimentally tested and validated.

In this study, we investigated the bacterial diversity associated with *Acer pseudoplatanus* L. trees growing around a TNT spill basin in a forest soil. Two TNT polluted soils that differed in the level of pollution and soil type were analyzed and compared to a non-polluted soil as reference. We used amplified ribosomal DNA restriction analysis (ARISA)-fingerprinting to assess the community structure of the resident bacterial communities and performed isolation of the characteristic microbiota. We performed 16S rRNA gene characterisation, molecular, enzymatic and phenotypic characterisation of their plant growth promoting and TNT-transformation abilities. In addition, *Agrostis capillaris* L. was used as plant-model to study PGP effects of selected isolates in a consortium under TNT stress.

## Materials and Methods

### Site Description and Sampling

Samples were collected at a military facility in Zwijndrecht, Belgium (51°11′40.0″N; 4°19′29.6″E) on July 8th 2013. Nine young *A. pseudoplatanus* trees were sampled, three from the lower TNT wastewater basin area (LT-site), three from the deeper forest (HT-site), and three from a non-polluted location (C-site). Rhizosphere soil that was held tightly by the roots after shaking to remove loosely adhered soil, was collected. Bulk soil samples (10 kg soil per sample) were taken adjacent to each tree; namely the layer between −10 cm to −30 cm was collected after manually removing the mulch and top-soil-layer. Soil type was sandy-loam (USDA classification), average pH of 6.2 ± 0.8, variable labile organic C-content (HT: 3074 ± 152 mg kg^−1^; C: 1625 ± 15 mg kg^−1^; LT: 1079 ± 28 mg kg^−1^), and variable inorganic nitrate content (HT: 17 ± 1.2 μg g^−1^; LT: 5.6 ± 0.8 μg g^−1^; C: 3.8 ± 0.2 μg g^−1^). TNT-concentrations in the bulk soils were 47 911 ± 1001 mg TNT kg^−1^ at HT and 3021 ± 435 mg TNT kg^−1^ at LT, and 0 for the non-polluted site (limit of detection, 0.2 μg kg^−1^). TNT-concentrations in the *A. pseudoplatanus* rhizospheres were 397 mg kg^−1^, 97 mg kg^−1^ and 0 mg kg^−1^ for HT, LT and non-polluted location respectively. Upon arrival to the lab, all samples were processed for culturing on the same day and aliquots for DNA-extraction were frozen at −80°C after homogenization by sieving (2 mm). Remaining soil was refrigerated (4°C) in the dark before physicochemical soil analysis.

### DNA-Extraction

DNA was extracted using the PowerSoil DNA extraction kit (MO-BIO, Laboratories, Carlsbad, CA, United States) according to manufacturer’s instructions. The grinding step was optimized to a 10 min shredding at 65 hertz using a Retsch MM2000 grinding mill (MA, United States) to recover the most quantity of high-molecular weight DNA. DNA integrity and purity were checked using agarose gel-electrophoresis and spectrophotometry using a NanoDrop (NanoDrop Technologies Inc., Wilmington, DE, United States).

### Automated Ribosomal Intergenic Spacer Analysis (ARISA)

ARISA was performed using the primers ITSF (5′-GTCGTAACAAGGTAGCCGTA-3′) and ITSReub (5′-GCCAAGGCATCCACC-3′), which amplify the 16S-23S rRNA intergenic transcribed spacers, using 1–5 ng/μl of DNA as input, the high-fidelity PCR kit of Roche (FHIFI ROCHE, Vilvoorde, Belgium) and PCR program as described (Cardinale et al., 2004). PCR-products were separated using a DNA-1000 chip and 2100 Bioanalyser (Agilent Technologies, Diegem, Belgium). The acquired ARISA data profiles were processed using StatFingerprints in R^1^ with data normalization and background subtraction.

## Cultivable Bacteria Isolation

One gram of soil sample was shaken for 1 h in 10 mM phosphate-buffer (per liter: 2.36 g Na_2_HPO_4_; 1.80 g NaH_2_PO_4_, 85.0 g NaCl and 200 μl tween 80; pH 6.8), and 100 μl of a 5-fold dilution series was spread on media plates. The selected media comprised: modified 284 minimal medium (Schlegel et al., 1991) supplemented with 200 μl of the organic carboxylic acid mixture EXU ROOT^®^ per liter (Innovak Global, Chihuahua, Mexico), Pseudomonas agar base CM0559 with Pseudomonas CN selective supplement SR0102 (Oxoid Limited, Hampshire, United Kingdom), BBL^TM^ Columbia CNA agar (BD Benelux, Erembodegem, Belgium) with 5% sheep blood for Gram-positive bacteria, and 1/10 869 rich medium for general heterotrophes (Mergeay et al., 1985). At regular time intervals during incubation, colonies were picked, purified and stored at −45°C, in a 15% w/v glycerol with 0.85% w/v NaCl solution. For biolog ECOplate metabolic profiling, the 10^−2^ dilution was used and soils were inoculated in triplicate on each 96 well plate (31 different carbon and amino sources ^2^ ). Absorbance was measured at selected time intervals at 590 nm using the FLUOstar Omega Microplate reader (BMG Labtech, Isogen Life Sciences, Temse, Belgium).

### Genotypic Identification of Cultivable Bacteria

DNA was extracted from all isolates using a DNeasy Blood and Tissue kit (Qiagen, Venlo, Netherlands) and typed by 16S Sanger sequencing using the universal prokaryotic 1392R primer (5′ ACGGGCGGTGTGTRC 3′) and the bacteria-specific 26F primer (5′ AGAGTTTGATCCTGGCTCAG 3′) with PCR-conditions as previously described (Barac et al., 2004). The sequences were quality trimmed using Geneious v4.8.5 and classified using the Ribosomal Database Project tool (Wang et al., 2007).

### Functional Assays of Cultivable Bacteria

For the TNT-transformation studies, we used a minimal salts medium previously described by Snellinx et al. (2003) consisting of 50 mM phosphate/NaCl buffer (pH 6.8), trace elements, 53 mg l^−1^ NH_4_Cl and 0.3% (w/v) glucose as carbon source (Snellinx et al., 2003). TNT was added after autoclaving to a concentration of 113.56 mg l^−1^ (500 μM) using a stock solution in DMSO (0.5% w/v). For solid media, 15 g Noble agar (BD Diagnostic Systems, Erembodegem, Belgium) was added per liter.

#### Nitroreductase Enzyme Activity

Bacteria were precultured in standard rich medium (869) at 30°C for 24 h on a shaker (Greiner Bio-One, Wemmel, Belgium), washed and resuspended in sterile 10 mM MgSO_4_ pH 7.0 to an OD_600__nm_ of 1. Then, 10 μl was transferred to microplate wells (Greiner Bio-One, Wemmel, Belgium) containing 150 μl minimal salts medium supplemented with 113 mg l^−1^ μM TNT, 53 mg l^−1^ NH_4_Cl and 0.3% w/v glucose. The microplates were incubated for 24 h at 30°C under aerobic conditions on a shaker. After 24 h, the reduction of TNT to nitro-reduction products was judged from the orange-yellow colouration of the growth medium by visual scoring. In addition, TNT was assayed by a colorimetric method based on the formation of Meisenheimer complexes after reaction of TNT with sodium sulfite at high pH (Jenkins and Walsh, 1992). Briefly, to 20 μl sample, 180 μl acetone and 20 μl TNT-solution (2 g sodium sulfite and 10 pellets KOH in 10 ml dH_2_O) was added and the plate was immediately shaken vigorously. After centrifugation, the absorbance of the supernatants was measured at 540 nm and TNT concentration calculated based on a standard curve prepared from 0 to 500 μM TNT. A red color indicates TNT, colorless TNT reduction products. A score of 1 was given for complete TNT-reduction, 0.5 for incomplete reduction (pink color), and 0 when no TNT reduction was observed. Additionally, nitrite released from TNT was measured using the Griess reagent system (Griess, 1879).

#### Metabolic Versatility

Bacteria were grown on overnight, resuspended in sterile 10 mM phosphate buffer (10 mM; pH 6.8) to an OD_600_ of 1. Then, 0.5 ml of each strain belonging to the same phyla or Proteobacteria class were mixed together to generate 6 consortia (Actinobacteria, Bacteroidetes, Firmicutes, Alphaproteobacteria, Betaproteobacteria, Gammaproteobacteria, *n* = 10 strains per consortium). Cultures were diluted 10^−2^ in phosphate buffer and incubated at 4°C overnight. Next, 150 μl of each diluted consortium was inoculated in each well of a Biolog ECO plate and incubated at 30°C for 5 days (*n* = 3 replicates). Absorbance was measured as described above.

#### *In vitro* Plant Growth-Promoting Activity

Bacteria were grown overnight, washed and resuspended in 2 ml sterile MgSO_4_ solution to obtain a suspension with OD_600_ of 0.5. Twenty microliter of this suspension was used for the inoculation of 96-well microplate assays (Greiner Bio-One, Wemmel, Belgium) for detection of: auxin production using the Salkowski reagent method (Patten and Glick, 2002); siderophore release was determined by using the Chrome azurol S (CAS) assay (Schwyn and Neilands, 1987); 1-aminocyclopropane-1-carboxylate (ACC)-deaminase activity was estimated by monitoring the amount of α-ketobutyrate generated by the enzymatic hydrolysis of ACC, a precursor of the plant hormone ethylene (Belimov et al., 2005); organic acid production was determined using the organic dye Alizarin red S (Cunningham and Kuiack, 1992), and acetoin production, measured using the Voges–Proskauer assay (Romick and Fleming, 1998). Bacterial chitin solubilisation was evaluated by dropping 10 μl of the bacterial suspension on plates with colloidal chitin, prepared from crab shell chitin (Sigma, Gent, Belgium) as described elsewhere (Hsu and Lockwood, 1975; Uroz et al., 2013). After incubation at 25°C for 7 days, the clearing of the initially turbid medium indicated chitin hydrolysing bacterial isolates. For all the PGP-assays, the bacterial isolates were distributed into classes scored as ‘0’ and ‘1’ depending on their negative or positive responses.

### Pot Experiment

Common bent (*A. capillaris*) seeds were surface sterilized and sown at a density of 150 mg seeds per 200 g sand on sterile autoclaved fine-grade sand spiked with 0, 25, or 50 mg TNT kg^−1^. Sterilized sand and seeds were used to eliminate the effects of indigenous TNT-degrading bacteria, and sand was used to reduce the probability of covalent binding of TNT to soil organic matter and clay particles. TNT was dissolved in acetone and mixed with the sterilized sand. Plants were watered periodically with 1/10 Hoagland solution (Hoagland and Arnon, 1950) and placed in a greenhouse with the following conditions: photoperiod of 14:10 h light:dark, a temperature cycle of 22°C day:18°C night and a relative humidity of 57%. After establishment of the primary roots (1 week after germination), the plants were inoculated by sand drench with a bacterial consortium in autoclaved 10 mM MgSO_4_ at a concentration of 10^6^ cells g^−1^ sand, while non-inoculated plants were watered with the same amount of sterile MgSO_4_. All pots were replicated in eight pots per condition, no plant controls, no TNT controls, and no inoculation controls were taken along the experiment. From 4 weeks old plants, biomass, root and shoot length were determined. Acetonitrile-extractable TNT-concentrations in the sand substrate were analyzed by HPLC as described (Thijs et al., 2014a). For the analysis of TNT in plant fractions, 1 g dried root or shoot material was crushed into a fine powder and extracted three times with 10 ml methanol before HPLC analyses. Colony forming units (CFUs) were determined according to standard procedures (Feng et al., 2002). For determining the activities of the antioxidative enzymes, 100 mg of root and 100 mg leaf tissue were collected and snap-frozen in liquid nitrogen. The activities of superoxide dismutase, glutathione reductase, glutathione-S-transferase, malate dehydrogenase, catalase and guaiacol peroxidase were determined spectrophotometrically as described previously (Thijs et al., 2014a).

### Accession Numbers

All partial 16S rRNA gene sequences of cultivable isolates were submitted to NCBI Genbank and are available under accession numbers MH337876 - MH338035.

## Results

### Soil Microbial Communities Diverge Depending on TNT Concentration

**FIGURE 1:**
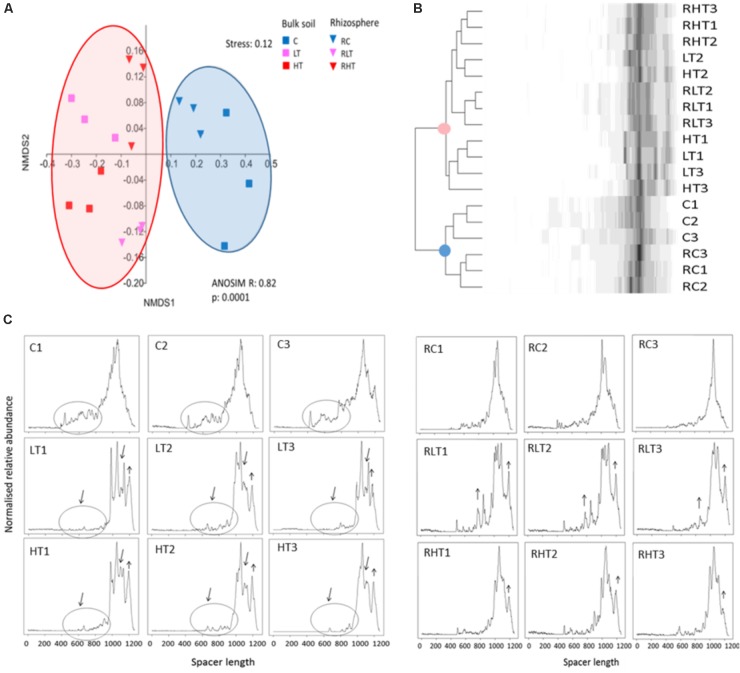
Total microbial communities in polluted soil samples are different from control samples. **(A)** Multivariate analyses of the Bray-Curtis matrix from ARISA profiles. **(B)** Dendrogram of the Bray-Curtis matrix and complete clustering method. **(C)** ARISA electropherograms of bulk soil (left) and rhizosphere (right) with in the X-axis the spacer length (in bp) and in the Y-axis the normalized relative abundance. Sample abbreviations: C, control, LT, low TNT, HT, high TNT location, R, rhizosphere, *n* = 9 samples per condition.

To obtain initial insights in the total bacterial community structure and diversity, we used automated fingerprint analyses. This revealed that the soil microbiomes of the TNT-polluted samples were significantly different from non-polluted samples (**Figure 1A**) confirmed by the Bray-Curtis cluster analysis (**Figure 1B**). The community shift and reduced diversity can also be observed in the ARISA-electropherograms (**Figure 1C**). In particular, a lower number of peaks corresponding to spacers in the range of 500–1000 bp was observed in the TNT-polluted bulk soils compared to the non-polluted bulk soils. In addition, the broad peak ranging from 900 to 1200 bp in the non-polluted bulk soil was gapped for the low and high TNT-polluted bulk soils, suggesting a diversity decrease. In contrast, spacers of 1200 bp long were increased in polluted samples compared to the non-polluted samples. Associated with these shifts, we recorded a significantly reduced Shannon diversity in polluted bulk soils (LT, 5.9 ± 0.05; HT, 5.8 ± 0.2 vs. C 6.4 ± 0.2) along with significantly reduced evenness in the low TNT-polluted bulk soil (LT, 0.36 ± 0.02 vs. C, 0.54 ± 0.03) (ANOVA, Tukey-HSD, *p* < 0.05). Shannon diversity and evenness were not different between the polluted and non-polluted rhizosphere samples as detected by ARISA. A comparison between bulk and rhizosphere samples, showed that rhizosphere samples were more diverse than the respective bulk soils, (ANOVA, Tukey-HSD, *p* < 0.05).

### SYCAM Culture Collection: Isolation and Genotypic Identification

**FIGURE 2:**
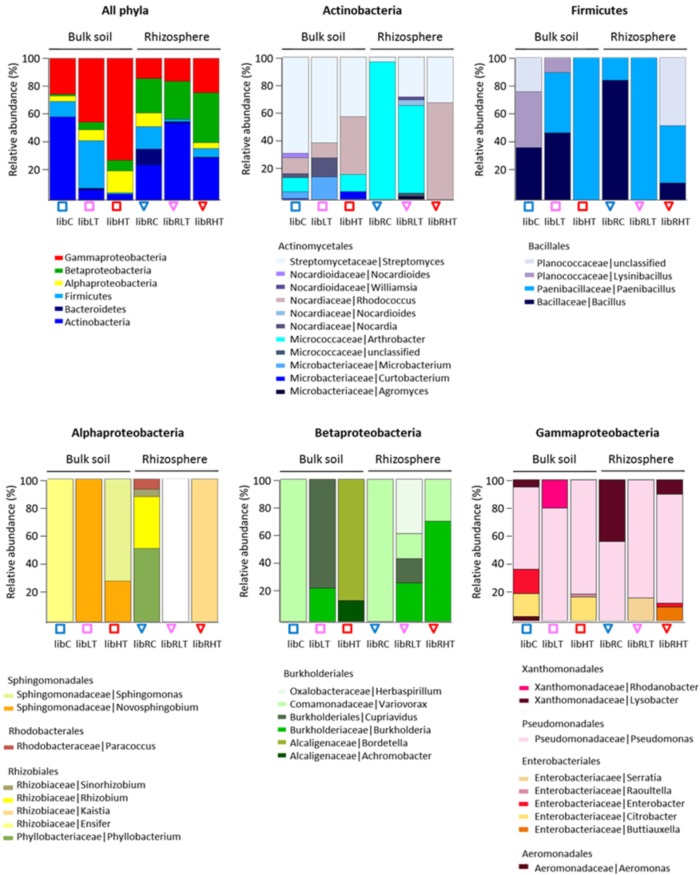
Overview of the genotypic identification and distribution of the isolated CFUs. Phyla and Proteobacteria class level are shown and the distribution of families present among the Actinobacteria, Firmicutes, Alphaproteobacteria, Betaproteobacteria, and Gammaproteobacteria.

Three hundred morphologically distinct colonies were picked from the plates and sub-cultured, from here on referred to as the SYCAM culture collection. The cultivable subsets were designated ‘Lib’, for library of CFUs followed by their sampling location identifier. Of these 300, a total of 160 different strains were detected by restriction fingerprinting, and all these were genotyped using 16S rRNA gene Sanger sequencing (see Supplementary Table S1 for an overview of the sequenced isolates per library). All of the sequences fell into four phyla of which 56% belonged to Proteobacteria, 31% to Actinobacteria, 11% to Firmicutes and 2% to Bacteroidetes (**Figure 2**).

The polluted bulk soils were dominated by the Proteobacteria, while the non-polluted bulk soil community was dominated by Actinobacteria followed by Gammaproteobacteria, Firmicutes and Alpha- and Beta-proteobacteria. In the rhizosphere, Actinobacteria were enriched in all three soil types with the highest proportion in the low TNT polluted rhizosphere (**Figure 2**). In addition, Proteobacteria, Firmicutes and Bacteroidetes were detected in the rhizosphere. Interestingly, there was a significant correlation (rs 0.92, *p* < 0.001) between the TNT concentrations in soil and the number of cultivated Gammaproteobacteria, specifically the genus *Pseudomonas*.

Isolated CFUs from the phylum Actinobacteria were exclusively Actinomycetales, of which the genus *Streptomyces* dominated in LibC and LibLT, while *Arthrobacter* was the dominant genus in the other libraries except for LibRHT, which was dominated by *Rhodococcus* (**Figure 2**). Betaproteobacteria isolates belonged entirely to the Burkholderiales and were represented only by the genus *Variovorax* in LibC and LibRC, whereas in the polluted soils *Burkholderia*, *Bordetella* (Alcaligenaceae), *Herbaspirillum* and *Achromobacter* were also detected. The Alphaproteobacteria isolates fell into the families Phyllobacteriaceae, Rhizobiaceae, Rhodobacteraceae, and Sphingomonadaceae in LibC, while only 2 families dominated the other libraries. CFUs of the phylum of Firmicutes belonged entirely to the Bacillales.

Counting of the bacterial CFUs on minimal 284 medium revealed that the non-polluted bulk soil contained 3.5 × 10^6^ bacterial CFUs g^−1^ DW soil, whereas the low TNT polluted bulk soil contained a significantly reduced number of cells (2.05 × 10^5^ CFUs g^−1^ DW soil) (ANOVA, Tukey-HSD, *p* < 0.05) (Supplementary Figure S1). The highest number of CFUs was obtained for the rhizosphere samples and there was no difference between non-polluted and TNT-communities (av. 5.2 × 10^6^ CFUs g^−1^ DW soil). Enumeration on selective *Pseudomonas* agar showed a significant increase in the number of Pseudomonads in the TNT-polluted bulk soils (LT, 8.6 × 10^6^; HT, 1.04 × 10^7^ CFUs g^−1^ DW soil) compared to the non-polluted bulk soil (4.8 × 10^6^ CFUs g^−1^ DW soil), supporting the results from the pyrosequencing dataset. In contrast, culturing on Gram-positive CNA agar revealed a significant reduction in the number of Gram-positive bacteria in both TNT-polluted bulk soil communities (LT, 2.05 × 10^5^; HT, 1.5 × 10^5^ versus C, 2.27 × 10^6^ CFUs g^−1^ DW soil).

### Metabolic Versatility and Plant Growth Promotion Potential

**FIGURE 3:**
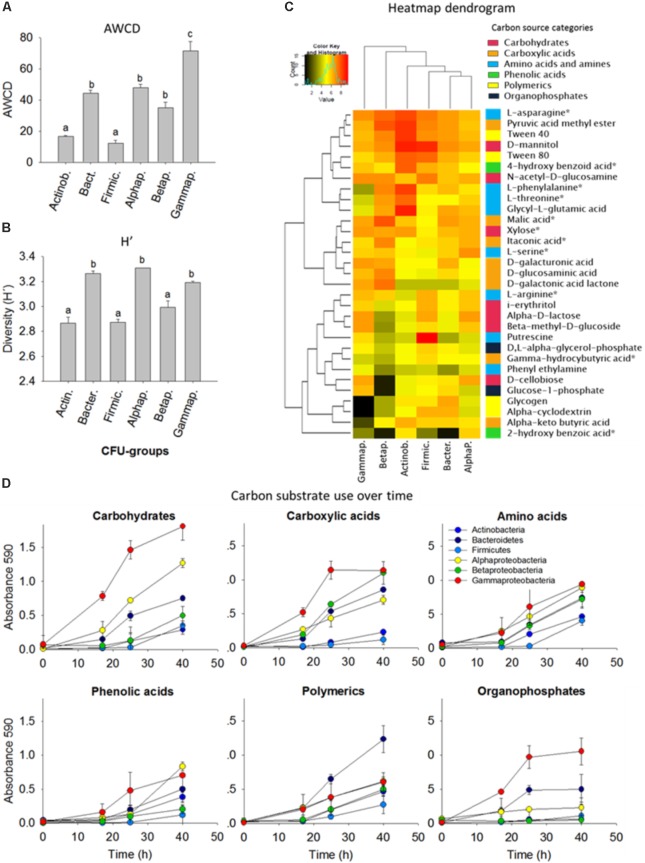
Comparison of carbon substrate utilization activities of isolated colony forming units (CFUs). **(A)** Average well color density (AWCD) (mean ± standard error bars) for carbon substrate utilization. **(B)** Diversity of carbon substrate use as judged by the Shannon index (H’). Different letters (a,b,c) indicate that the means are significantly different (ANOVA, Tukey HSD, *p* < 0.05, *n* = 3). **(C)** Heatmap dendrogram of the carbon substrate utilization based on the area under the absorbance versus time curve AAT/AWCD. Columns represent the bacterial phyla, in rows are the 31 carbon sources. The colored side-bar shows the categories to which the carbon sources belong. Carbon sources with an asterisk are common root exudates. **(D)** Carbon substrate use over time. 10 isolates were grouped per phyla/class and included for the Actinobacteria (*Arthrobacter*, *n* = 3; *Curtobacterium*; *Microbacterium*; *Nocardia*; Nocardioides; *Rhodococcus*, *n* = 2; *Streptomyces*), Bacteroidetes (*Chryseobacterium*, *n* = 3; *Dyadobacter*; *Flavobacterium*, *n* = 2; *Mucilaginibacter*), Firmicutes (*Bacillus*, *n* = 4; *Lysinibacillus*, *n* = 2; *Paenibacillus*, *n* = 3; Planococcaceae), Alphaproteobacteria (*Ensifer*; *Kaistia*; *Novosphingobium*; *Paracoccus*; *Phyllobacterium*; *Rhizobium*; *Sinorhizobium*; *Sphingobium*, *n* = 3), Betaproteobacteria (*Achromobacter*; *Bordetella*; *Burkholderia*, *n* = 3 *Cupriavidus*; *Herbaspirillum*; *Variovorax*, *n* = 3), and Gammaproteobacteria (*Aeromonas*; *Enterobacter*, *n* = 2; *Lysobacter*, *n* = 2; *Pseudomonas*, *n* = 4; *Raoultella*).

To assess the metabolic versatility of the bacterial strains, the isolates were grouped as 10 isolates per taxon and six different consortia were composed (Actinobacteria, Bacteroidetes, Firmicutes, Alphaproteobacteria, Betaproteobacteria, and Gammaproteobacteria, see **Figure 3**). Then the bacterial assemblages were separately inoculated into Biolog ECO plates to obtain the carbon-metabolic fingerprint.

Based on the Shannon H’ entropy (*p* < 0.05) the Alphaproteobacteria, Gammaproteobacteria, and Bacteroidetes were the most metabolically versatile of the cultured isolates; they could use several carbohydrates, carboxylic acids, amino acids, phenols and polymers including many of the common plant root-exudates (**Figure 3B**). Gammaproteobacteria showed overall the most rapid growth increase in response to carbon substrates and this was also indicated by the high AWCD value (*p* < 0.05). Firmicutes and Betaproteobacteria showed the lowest AWCD values illustrated by a slow growth after addition of carbohydrates and carboxylic acids (**Figure 3**). Alpha-, Beta- and Gamma-proteobacteria and Bacteroidetes were rapid consumers of small aromatic compounds, which can be released upon degradation of lignin, an important polymer in forest ecosystems. *N*-acetyl-D-glucosamine, the monomeric component of chitin, a major component of fungal cell walls in soils, was utilized by all groups (**Figure 3C**).

**FIGURE 4:**
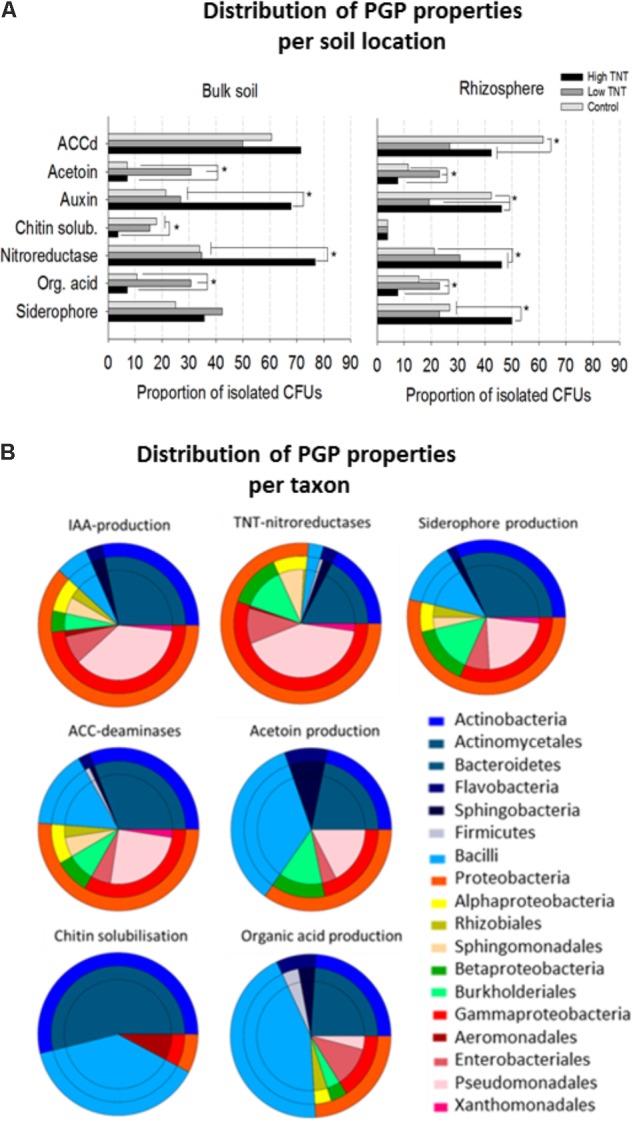
Proportion of bacterial isolates with plant growth-promoting features, nitroreductase activity and their distribution according to origin **(A)**. Comparison of the proportions of bacterial isolates exhibiting *in vitro* PGP-activities and nitroreductase activity. For each assay, all the cultivable isolates per library were tested. Significant differences in activities are marked with an asterisk (Fisher’s exact test for pairwise comparisons and FDR correction, *p* <0.05). **(B)** Pie diagrams showing the cumulative number of taxa with PGP and nitroreductase activity for each category as indicated above the pies. The outer rings represent the phyla and these are further broken down (inner rings) to the order level. For each test (*n* = 10 bacterial isolates of the same genus were included).

All isolates were further evaluated for a diverse set of plant growth promotion features including siderophores production, organic acids production, acetoin synthesis, indole-3-aceteic acid (IAA) and ACC-deaminases, chitin solubilisation potential, and nitroreductase activity. Positive PGP characteristics were distributed over many and a wide variety of the isolates (**Figure 4**), though major differences in relative abundance of certain traits was observed depending on their origin. For example, there was a significant enrichment of nitroreductase producing strains in libHT versus libC (**Figure 4A**). Auxin production can be said to be co-enriched with nitroreductase activity based on the Pearson coefficient of 0.94 (**Figure 4B**). LibLT was enriched for strains producing organic acids and acetoin, which also seem to be highly correlated (Pearson coefficient 0.95). Chitin solubilisation was more abundant in the non-polluted and low polluted bulk soils compared to the high polluted soil.

As with the bulk soil, an enrichment of nitroreductases in the highly polluted rhizospheres was found compared to the non-polluted rhizospheres and low polluted rhizospheres (**Figure 4B**). Auxin and siderophore production were also enriched in libRHT, while acetoin and organic acid production were more enriched in libLRT. Comparison of the rhizosphere fractions with the surrounding bulk soil indicated a higher abundance of chitin solubilizing isolates in the bulk soil and auxin producers in the rhizosphere of the non-polluted soil. Siderophore producing isolates were more enriched in the rhizosphere compared to the bulk soil for HT.

Inspection of the taxonomic distribution to which the isolates belong revealed that TNT-nitroreductase was found mainly in the Gammaproteobacteria group, in particular *Pseudomonas*, next to other plant growth-promoting rhizobacteria (PGPR) including *Enterobacter, Burkholderia, Sphingomonas*, and *Rhodococcus* (**Figure 4B**). The ability to produce auxin was found among the genera *Pseudomonas, Rhodococcus, Arthrobacter, Sphingobium, Bacillus*, and *Enterobacter*. The capacity for organic acid production and acetoin synthesis was associated most often with Firmicutes including *Bacillus* and *Paenibacillus* and further among Actinobacteria, in particular *Streptomyces*, and the Proteobacteria, specifically *Pseudomonas* and *Burkholderia*. Siderophore production and the presence of ACC deaminases were detected for a diverse group of isolates. In contrast, the potential to solubilize chitin was restricted to specific genera including *Streptomyces*, *Bacillus*, and *Aeromonas*.

### Characterization of Trinitroaromatic Compound Degraders

To efficiently screen the collection of cultivable isolates for their ability to transform TNT, as a model for trinitroaromatic compounds, a 96-well microplate assay was developed. The assay involves growing pure bacterial cultures separately in individual wells containing minimal medium with TNT (113 mg l^−1^), NH_4_Cl as a source of nitrogen (53 mg l^−1^), and 0.3% glucose as a source of carbon and energy. Nitroreductase activity was detected based on changes in the color of the medium. The conversion of the TNT-medium from colorless to yellow was indicative for reduction of the nitro-group of TNT resulting in the formation of aminodinitrotoluene (ADNTs) and diaminonitrotoluene (DANTs) (van Dillewijn et al., 2008a,b) (Supplementary Figure S2). In addition, production of a brown-orange color can indicate hydrogenation of the aromatic ring to hydride and dihydride-Meisenheimer complexes (Wittich et al., 2008) (Supplementary Figure S2 and Supplementary Table S2).

Of all the isolates tested, the Gammaproteobacteria, in particular the genera *Citrobacter, Enterobacter*, *Pseudomonas, Raoultella* and *Serratia*, and some members of the Alphaproteobacteria mainly *Sphingobium* spp., were found to grow rapidly in the presence of TNT (Supplementary Tables S1, S2). This was observed from the orange-colored pigments, presumably caused by Meisenheimer complexes formation, after 4–6 h of incubation and the accumulation of yellow amino reduction products (Supplementary Figure S2) after 24 h of growth. Isolates of *Burkholderia* and *Variovorax* (Betaproteobacteria) as well as *Novosphingobium* and *Kaistia* (Alphaproteobacteria) demonstrated slow growth in the presence of TNT and were able to partially reduce TNT, with 50% of the TNT remaining at the termination of the assay (Supplementary Table S1). Members of the genus *Rhodococcus* turned the medium to an orange color and accumulated nitrite in the supernatants, suggesting the production of Meisenheimer and hydroxylamino-derivates. The Rhodococci failed, however, to grow with TNT or nitrite (1 mM NaNO_2_) as sole N-source, and 80% of the TNT remained in the medium after 24 h (data not shown). No growth in the presence of TNT or no effective TNT-transformation were observed for the gram-positives *Arthrobacter*, *Microbacterium* and *Streptomyces* isolates (Actinobacteria), and for Bacilli and Paenibacilli (Firmicutes).

### Functional Performance of a Consortium of Selected PGP-Isolates for TNT Transformation in a Pot Experiment

**FIGURE 5:**
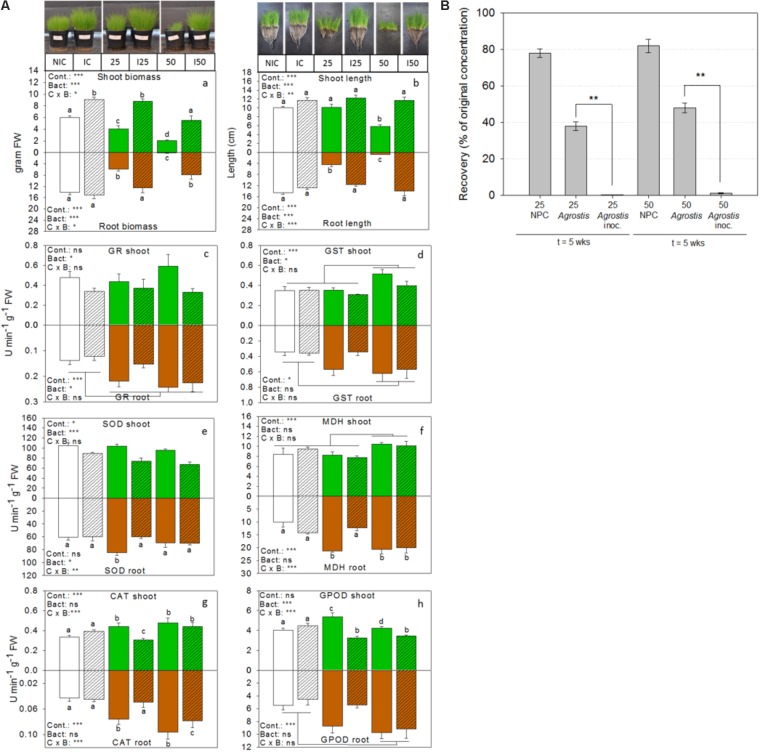
Bioaugmentation with consortium ST1 in the rhizosphere of common bent grass increases biomass, removes TNT and increases plant health. **(A)** Bar graphs showing plant biomass, shoot and root length, and activities of enzymes related to antioxidative defense for inoculated (I) TNT-exposed plants, inoculated non-exposed plants (IC) and non-inoculated non-exposed control plants (NIC). Shoot responses are shown in green (white for the control) and belowground root responses are shown in brown (white for the control). Bacteria inoculation conditions are shown in hatched bars. Enzyme activities are expressed in units per min and gram fresh weight. TNT exposure concentrations were 25 and 50 mg TNT per kg dry weight sand. Values represent average and standard-error of 8 biological replicates per treatment, ^∗^*p* < 0.05; ^∗∗^*p* < 0.01; ^∗∗∗^*p* < 0.001; ns, not significant. Cont., control; Bact., bacteria; C × B, contamination x bacteria interaction effect (two-way ANOVA, Holm–Sidak, *n* = 8). **(B)** Percentage of TNT remaining in the substrate after 5 weeks of growth. GR, glutathione reductase; GST, glutathione transferase; SOD, superoxide dismutase; MDH, malate dehydrogenase; CAT, catalase, GPOD, guaiacol peroxidase.

Among the CFUs isolated from the TNT polluted bulk soils and rhizosphere, several representatives were shown to possess multiple PGP and TNT-reductase activities interesting for rhizoremediation (Supplementary Table S1 and **Figure 4**). To assess the growth-promotion potential of a selection of these bacteria *in planta*, we inoculated the roots of non-exposed and TNT-exposed *Agrostis capillaris* seedlings with a 12-member consortium of isolates from bulk soil and *A. pseudoplatanus* rhizosphere of the TNT polluted location. This consortium consisted of: *Rhocococcus* sp. zw191, *Kaistia* sp. zw161, *Novosphingobium* sp. zw55, *Burkholderia* sp. zw160, *Cupriavidus* sp. zw211, *Herbaspirillum* sp. zw98, *Variovorax* sp. zw90, *Raoultella ornithinolytica* TNT(/zw146), *Pseudomonas* sp. zw94, zw89 and zw38. *Agrostis* was used in this experiment because it shows vigorous growth, has a dense rooting system and is a common grass species on the military sites in Belgium. After 5 weeks, the growth of non-inoculated TNT-exposed plants was significantly inhibited (biomass and root and shoot length) compared to non-exposed control plants, whereas plants inoculated with the bacterial consortium showed a significant improvement of their growth (ANOVA, Holm–Sidak, *p* = 0.0001, *n* = 8) (**Figure 5**). In particular, non-inoculated plants exposed to the highest TNT-concentration (50 mg TNT kg^−1^) had poorly developed roots (**Figure 5**), while inoculated plants showed a 200–400% increase of both, root weight and length. Differences in root length can be explained by the inoculant used (F:145.1, *p* = 0.001), an interaction term (F:69.6, *p* = 0.001) and pollution level (F:57.1, *p* = 0.001). Differences in root mass were largely explained by a pollution effect (F:49.0, *p* = 0.001) and an inoculum effect (F:26.1, *p* = 0.001). As with the roots, shoot weight of inoculated and TNT-exposed plants doubled compared to non-inoculated TNT-exposed plants. Differences in shoot weight were explained by a bacteria effect (F:255.6, *p* = 0.001) and by pollution (F:93.1, *p* = 0.001).

In addition, we determined the activities of six enzymes involved in cellular defense against oxidative stress in the roots and shoots of TNT-exposed and unexposed plants (**Figure 5A**). A two-way ANOVA for the effect of the treatments on enzyme activity patterns revealed a dominant bacterial effect (F:11.2, *p* = 0.0001), a pollution effect (F:4.4, *p* = 0.0002), and interaction effect (F:3.7, *p* = 0.002), suggesting a differential effect of the bacteria in polluted and non-polluted substrate. Bacterial inoculation explained the lowered glutathione reductase (GR)-activity in the shoots (F:5.8, *p* = 0.02) compared to non-inoculated TNT-exposed plants, whereas a dominant pollution effect explained the high GR-reductase activity in the roots (F:12.2, *p* = 0.0004). Differences in glutathione transferase (GST) activity in shoots and roots were also explained by pollution level (F:7.8, *p* = 0.001 and F:3.8, *p* = 0.03). In particular, GST activity was significantly increased in the highest TNT-exposed plants. Differences in malate dehydrogenase activity in the shoots were influenced by pollution level (F:12.8, *p* = 0.0001), while in the roots, both the effect of pollution (F:11.8, *p* = 0.0001) and the interaction term (F:9.5, *p* = 0.0003) were significant. A dominant bacterial effect (F:38.2, *p* = 0.0001) lowered the superoxide dismutase (SOD) activity in the shoots of TNT-exposed plants relative to the non-inoculated ones, while in the roots both the interaction term (F:7.9, *p* = 0.002) and the bacteria effect (F:4.4, *p* = 0.03) were significant. The activity of catalase in shoots and roots was explained by a significant interaction effect (F:10.8, *p* = 0.0006 and F:10.7, *p* = 0.0006) and pollution effect (F:6.5, *p* = 0.006 and F:6.4, *p* = 0.005). Guaiacol peroxidase (GPOD) activity in shoots was impacted by an interaction term (F: 13.9, *p* = 0.0001) and bacterial effect (F:19.1, *p* = 0.0002) but showed no pollution effect. In contrast, GPOD activity in the roots was enhanced by pollution (F: 6.07, *p* = 0.0006).

At the end of the experiment, extractable TNT-concentrations in the sand were determined (**Figure 5B**). Less than 1% TNT remained in the pots when *Agrostis* was inoculated with consortium ST1, whereas 35–48% of the initial applied TNT was recovered from the non-inoculated pots with *Agrostis*. Control pots without plants and without inoculum still contained more than 80% of original TNT-concentration. The recovery efficiency of TNT from the substrate was 90.1 ± 6.5%, the recovery of initial spiked TNT was 22.01 ± 0.3 mg TNT (for the 25 mg/kg condition) and 43.70 ± 0.7 mg TNT kg^−1^ (for the 50 mg/kg condition). The TNT-transformation products were only detected in inoculated conditions and only in trace amounts (in all <5%). Roots and shoots were also analyzed for the presence of TNT and its transformation products. TNT was retained in the roots of inoculated plants and only very low concentrations were detected (0.034 mg TNT per gram dry weight root in substrate amended with 50 mg TNT kg^−1^) and none in the shoots. This was similar for the non-inoculated plants. At the end of the experiment, the numbers of viable bacterial cells per gram rhizosphere were determined in the inoculated pots. These were respectively 2.3 × 10^5^ for the control, 4.32 × 10^5^ for the 25 mg TNT per kg sand conditions and 7.02 × 10^5^ CFUs for the 50 mg TNT per kg soil. Although this was a significant decrease compared to the initial number (10^6^ CFU g^−1^ sand), it was 100 times higher than the non-inoculated substrates (average <10^3^ CFU g^−1^ sand), suggesting rhizosphere colonization.

## Discussion

This study presents the isolation and detailed characterisation of 300 bacterial strains, called the SYCAM collection, from *A. pseudoplatanus* trees growing on a military site with a long history of explosives pollution. Because of this fact, the site represents a unique resource in terms of finding potentially novel strains with PGP and catalytic activities to be able to deal with multi-compound pollution in a hostile environment with low nutrients. Moreover, plants at such sites are expected to have more close association with their rhizospheric microorganisms, to be able to germinate and grow in polluted soil, in comparison to non-polluted habitats. Therefore, as a first confirmation of our hypothesis, we applied a rapid and inexpensive, though high-throughput, automatic ARISA fingerprinting technique, to get a glimpse of the total microbial communities and their differences or similarities across compartments and locations.

A clear shift was noticed in the bacterial community composition, with the main effect explained along the *x*-axis with TNT pollution and the secondary effect was the separation between bulk soil and rhizosphere, an effect which was much more pronounced for the polluted soils compared to the control site (**Figure 1**). Previous studies also found that TNT induces shifts in bulk soil bacterial communities based on PUFA, DGGE or microarray analysis (Fuller and Manning, 1998; Eyers et al., 2006; George et al., 2008; Travis et al., 2008). In this study, ARISA chip technology has the advantage to be more comparable between sample sets, and to give a higher reproducible result, because of the automatization, and at a fairly high resolution (bands of < 10 bp are distinguished) compared to conventional agarose or polyacrylamide gels. Though the advantage of DGGE compared to ARISA is the separation of isolates based on GC content, and therefore a unique band per strain is expected, whereas the 16S-23S rRNA intergenic spacer region is highly variable amongst strains, but can also be of similar length for very phylogenetically distant strains, so bands are not easily assigned to groups of bacteria (Schmieder and Robert Edwards, personal communication), which doesn’t preclude that specific primers targeting specific groups of interest can be used with ARISA, reducing the sample complexity. Though ARISA served still a very useful technique to discriminate diversity patterns and shifts even in a very rich forest soil (**Figure 1B**) and rhizosphere soil.

Dramatic decreases in community richness, Shannon and Simpson diversities were observed in the HT soil, indicating high toxicity experienced by the residing soil microbiome and potentially affecting their functioning. It was also obvious that TNT differentially affects diversity in the rhizosphere. TNT can get complexed with organic compounds exuded by the roots, and form amino-metabolites to humified organic matter, which can decrease its bio-availability and hence toxicity (Hundal et al., 1997; Kreslavski et al., 1999; Thorn et al., 2002). Though, active transformation by different microorganisms in the rhizosphere compared to the bulk soil, can also in part explain the lower TNT concentrations in the rhizosphere and feedback effect on the microorganisms.

As ARISA confirmed our initial expectations that this site harbored different microbial communities compared to a non-polluted forest site, the isolation and characterization of bacterial isolates was straightforward with the aim to obtain a genetic and cultivable pool of plant-associated strains, which may serve high purpose for bioremediation, or other biotechnologies. Our results indicated that using five different media, and especially the medium 284 with synthetic plant root exudates, was appropriate for the isolation of Gamma- and Beta-proteobacteria, Actinobacteria, Bacteroidetes and Firmicutes (**Figure 2**). A very diverse subset of genera was isolated such as *Curtobacterium*, *Kaistia*, *Ensifer*, *Sphingomonas*, *Bacillus*, *Paenibacillus*, *Pseudomonas*, *Actinobacterium*, *Agromyces*, *Aeromonas*, *Pseudomonas*, *Streptomyces*, many of which have previously been described as taxa holding plant growth promoting strains (Compant et al., 2010; Barret et al., 2011; Bruto et al., 2014). Although, the selection of the media did not govern the isolation of new phyla, the diversity of cultured phyla of the SYCAM collection can be expanded in future using additional complementary media, targeting other bacterial groups of interest, as done for the large scale *Arabidopsis* *thaliana* A*t*-SPHERE culture collection study (Bai et al., 2015).

Bacteria that have a high tolerance to TNT will be favored in TNT-polluted soils. In this study, Gram-negative bacteria were shown to be strongly enriched in the TNT-polluted soils while Gram-positive bacteria were depleted in both TNT-polluted bulk soils and the rhizosphere based on CFU-counts (Supplementary Figure S1). Previously, negative effects of TNT on Gram-positive bacteria have been reported and the underlying mechanisms yet remain to be determined (Fuller and Manning, 1997; Eyers et al., 2006). A hypothesis is a covalent cross-linking of cell membrane lipopeptides by TNT inhibiting cell replication (Ho et al., 2004; McBroom and Kuehn, 2007; Cho et al., 2009). Alternatively, gram-negative bacteria may deal with environmental pollution by enhancing the production of exopolymers, as observations for *Pseudomonas putida* HK-6 exposed to TNT show (Lee et al., 2008) or for example for *P. putida* KT2440, by increasing the expression of genes involved in detoxification (antioxidative response pathways, glutathione biosynthesis and nitroreductases) in addition to active efflux pumps to maintain low intracellular TNT-concentrations (Fernandez et al., 2009). Based on our findings of CFU count, it is suggested that the difference in sensitivity toward TNT will most likely affect the strategies used by the bacteria to transform and detoxify TNT, and explain the overall lower or higher fitness.

Several bacterial strains that originated from the polluted soil and rhizosphere (*Rhizobium*, *Burkholderia, Enterobacter, Pseudomonas*, *Sphingomonas*, *Raoultella*), were able to co-metabolically transform TNT to amino-reduction products (**Figures 3**, **4**). This is not completely unexpected as nitroreductases are fairly diverse distributed in bacteria (Roldan et al., 2008). Many of the bacterial isolated we recovered seem to be able to catalyze the aromatic ring reduction of TNT yielding monohydride- and dihydride-Meisenheimer products. Especially, the high proportion of *Pseudomonas* spp. with nitroreductase activity in the cultivable collection is highly interesting and demonstrates the usefulness of this strain in TNT-bioremediation. Nitrite was not at all times detected in the supernatants, probably indicating that it is consumed by some strains, as we have shown previously (Thijs et al., 2014b). The formation of Meisenheimer complexes, and diarylamines with the concomitant release of nitrite from TNT is an important metabolic route for TNT detoxification (van Dillewijn et al., 2008b; Wittich et al., 2008; Wittich et al., 2009). This pathway has garnered a significant interest for TNT biotransformation and detoxification (Stenuit and Agathos, 2010). This is also shown by the fact that some of these bacterial nitroreductases have been successfully engineered in plants which significantly improved plant survival, and increased phytoremediation effectiveness (Travis et al., 2007; Rylott et al., 2011a). Further experimental evidence of TNT detoxification and characterisation of the transformation products in many of our isolates are in the pipeline. As an example, the fully genome-sequenced and experimentally characterized isolate, *Raoultella ornithinolytica* strain TNT (Thijs et al., 2014b), was shown to hold the *N*-ethylmaleimide reductase gene, catalyzing TNT-denitration combined with nitrite consumption, only previously described for the laboratory model strains, *Escherichia coli* which lacks the genes to metabolize nitrite as N-source, *Enterobacter cloacae* (Bryant and DeLuca, 1991; Bryant et al., 1991), and *Pseudomonas putida* KT2440 (van Dillewijn et al., 2008a,b). This highlights the genetic potential of this culture collection. On a side, overall Gram-positive bacteria such as *Rhodococcus*, *Bacillus* and *Arthrobacter* did not show growth on TNT or efficient TNT reduction, which corroborates earlier studies (Fuller and Manning, 1997; Thijs et al., 2014a), and thus these taxa are not the candidates to look for in follow-up studies.

The high proportion of cultivable isolates with abilities to produce plant hormones (e.g., auxin, acetoin), release Fe (siderophores) and exhibit ACC-deaminase activity, which plays a role in decreasing ethylene-induced stress (Glick, 2005), indicates the bacteria being studied have adapted to live in close association with the plant host, and in a stressfull environment (**Figure 4**). Also, thirteen chitin-degrading bacteria of the Actinobacteria (*Streptomyces, Bacillus*) were isolated from the soil and rhizosphere samples. Chitinases produced by *Streptomyces* sp. have been shown to suppress the growth of phytopathogenic fungi (Hoster et al., 2005) which can be an interesting property for further research on antifungal activity. ACC-deaminase plays an important role in reducing plant ethylene-induced stress (Glick, 2014); this may explain part of the enhanced plant health effects seen in the inoculated treatments (**Figure 5**). The cultivable community of RLT showed an increased proportion of organic acid producing strains, dominated by Bacilli. Organic acid production by PGPR can improve phosphate solubilisation (Richardson et al., 2009; Bianco and Defez, 2010), which is a limiting plant nutrient, and may contribute to the complexation of toxic metal ions (Cu, Pb, Zn, Cd) (White et al., 1997).

Finally, to get insights into the mechanisms of PGP and TNT-detoxification by rhizospheric bacteria, community members of the low and high TNT-polluted rhizosphere subgroups were investigated in a rhizosphere-colonization experiment to verify if bacteria identified as members of the *Acer* rhizosphere and bulk soil communities were able to promote plant growth, and if bacteria were able to influence plant physiology at the level of their antioxidative defense. It is well known that TNT induces oxidative stress in plant cells leading to the over-production of reactive oxygen species (ROS) (Johnston et al., 2015). Activation of enzymes responsible for reduction, oxidation and conjugation of TNT such as nitroreductases, peroxidases, phenoloxidases and glutathione transferases has been reported in several plant species (Adamia et al., 2006; Brentner et al., 2008; Beynon et al., 2009; Cummins et al., 2011; Gunning et al., 2014). In 2015, researchers found that a mutation in the gene MDHAR6 encoding a monodehydroascorbate reductase offers enhanced TNT tolerance which is very promising, but these mutant lines have not been tested in combination with catalytic and plant-growth promoting consortia (Johnston et al., 2015). In addition, limited evidence exists about how bacteria can influence the activities of antioxidative enzymes in plants native to military locations (Rylott et al., 2011b). Our results showed that after 5 weeks of growth, 35–48% of the TNT remained in pots with the grasses that did not receive a bacterial inoculum, whereas less than 1% TNT was left in pots with grass that was inoculated with the bacterial consortium, suggesting active TNT-transformation by the bacteria in the rhizosphere. In addition, bacterial inoculation increased the above- and below-ground biomass of the plants exposed to 25 and 50 mg TNT kg^−1^ sand, reduced SOD and GPOD-activities in the leaves and affected, to some extent, GR- and GST-activities. Inoculated grasses showed indications of oxidative stress as observed from the elevated activities of antioxidative enzyme. For instance, concentration-dependent responses of catalase and guaiacol peroxidase activity were observed along with an increase in the activity of GST in root tissues. This dose-response relationship between GST and TNT was consistent with earlier observations for poplar trees exposed to TNT (Brentner et al., 2008). The correlation between GST and TNT can be explained by the phase three conjugation reactions according to the green liver model, whereby complexation with glutathione and storage in the central vacuole is used by the plant as detoxification mechanism (Sandermann, 1994). Malate dehydrogenase activity was also increased in response to TNT concentration and indicates increased flux through the tricarboxylic acid cycle, which generates reduced equivalents of NAD(P)H. While the increase of the antioxidative activity in roots may be explained by the local impact of TNT on cell structure and function, the stimulation of antioxidative enzymes in leaf tissues suggests that the presence of bacteria in the rhizosphere indirectly alters leaf physiology or results in the transmission of stress signals from root to leaves as observed for metal-stress (Opdenakker et al., 2012; DalCorso et al., 2013). In addition, this may also be explained by low levels of TNT transported to the aerial parts and stimulating gene expression in shoot and leaf tissues. Other papers have found as well that TNT is only limited transported to plant shoot tissues (Vila et al., 2007; Brentner et al., 2010). The observed increases in the shoot and root weights after inoculation may have resulted from pleiotropic bacterial effects such as detoxification of TNT in the rhizosphere, changes in plant nutrition, release of plant growth hormones or production of enzymes reducing stress-ethylene levels in the plant (ACC-deaminase) restoring a better balance between pro-oxidants and antioxidants in the plant. Together, these findings indicate that bacteria originating from the *A. pseudoplatanus* rhizosphere and bulk soil, play important roles in growth and survival of plants on TNT polluted soil. Subsequent extrapolation to the field is recommended to study the interactions in the most complex soil environment.

TNT rhizoremediation in natural soils is difficult and slow as a result of high TNT toxicity, abiotic limitations (e.g., bioavailability, heterogeneity of pollution, soil structure, pH and nutrients) and microbial competition. The observation of taxa-specific abundance shifts in the rhizosphere of TNT-soils and the high nitroreductase activity found amongst the cultured members, supports the hypothesis that natural TNT-rhizoremediation by *Acer* trees in the field is indirectly accomplished by the rhizosphere microbes. Hence, phytoremediation has to be considered as a promising strategy. To stimulate phytoremediation, inoculation of plant growth promoting and degradative strains may overcome some of the inherent constraints plants face when colonizing and growing in polluted soils. The isolation and functional characterisation efforts of cultivable members corresponding to important soil groups as *Pseudomonas* spp. with TNT-transforming activity are significant steps forward to reaching our ambition in enhancing the knowledge of plant-associated bacteria. A next useful step would be the evaluation of our strains not only for TNT transformation but also other toxic compounds, like PCBs, and on the other hand prospecting our SYCAM collection for potential antibiotic producing strains, CRISPR-cas loci, multi-strain interaction studies (Serrano-González et al., 2018) and metatranscriptomic analyses. CRISPR-cas engineering of either bacterial genes or the host plant enzymes, e.g., swapping more active gene variants for others, can be a future path in phytoremediation (Basharat et al., 2018). In all, combined metatranscriptomics and analytics will be particularly informative in unraveling the keys to plant growth-promotion and TNT-detoxification in the rhizosphere.

## Conclusion

In summary, the SYCAM bacterial culture collection holds 300 bacterial isolates with multiple PGP features and catalytic genes, originating from a unique location with high level nitroaromatics explosives pollution. Culture-independent analyses revealed a strong effect of TNT on microbial community composition and diversity in the forest soil, driving distinct communities. In these communities surviving in the harsh conditions, representative strains covering the main phylogenetic groups in soil and rhizosphere were recovered, purified and maintained in culture. Production of auxin, siderophores and the volatile hormone acetoin are a few of the traits multiple strains scored positive for, besides nitroreductase activity to detoxify TNT, but potentially also structural nitroaromatic homologues found in pesticides. Some of the strains in this culture collection have no genome sequenced representatives yet in the database, such as *Agromyces terreus*, and thus this again indicates the many potential novelties this collection holds, also in terms of catalytic functions. Hence, with this new strain repository, available for further phenotypic and *in planta* characterisation in addition to full genome sequencing, we aim to contribute to a better understanding of plant growth promotion, and plant microbiome functioning, to construct consortia which can enhance phytoremediation of the most recalcitrant compounds.

## Author Contributions

SoT, WS, NW, and JV participated in planning of research, interpretation of obtained results, and manuscript writing. SoT performed all experiments. SaT, BB, and PvD helped with interpretation of obtained results. PS and RC contributed with chemical analyses. JvH, as microbiologist expert, provided valuable feedback on figure layout and structuring of the Results section, and proofread the manuscript for English language.

## Conflict of Interest Statement

The authors declare that the research was conducted in the absence of any commercial or financial relationships that could be construed as a potential conflict of interest.

## Footnotes

1 http://cran.at.r-project.org

2 biolog.com
