# Supplementary material for: The Sycamore Maple Bacterial Culture Collection From a TNT Polluted Site Shows Novel Plant-Growth Promoting and Explosives Degrading Bacteria
Source: Front Plant Sci. 2018 Aug 3;9:1134. doi: 10.3389/fpls.2018.01134 (PMC6085565; doi:10.3389/fpls.2018.01134)
Supplement: Supplementary file 1 [file Table_1.DOCX]

**Supplementary figures and tables**


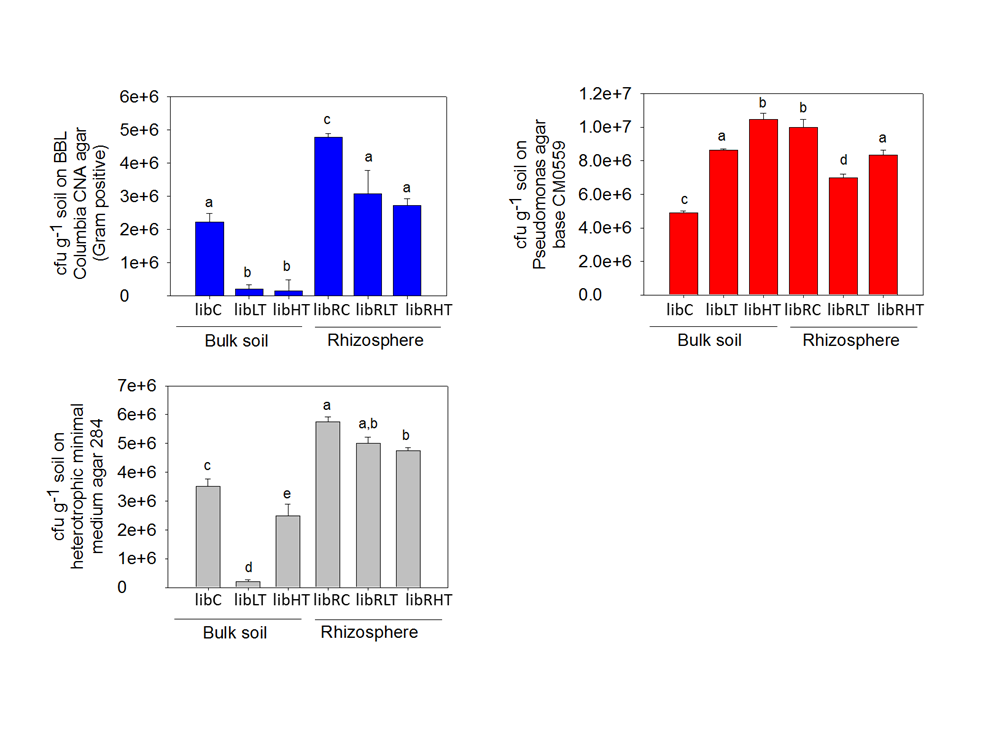


**Figure S1**: **Gram-positive CFUs decreased in TNT polluted bulk soils as well as total heterotrophs, but *Pseudomonas* sp. increase in relative abundance.** Bars represent CFU counts per gram of soil dry weight after 7 days of incubation on Gram-positive agar **a.** on Pseudomonas agar **b.** and on 284 minimal medium. Different letters indicate that the means are significantly different (ANOVA, Tukey HSD, p < 0.05, n=3).

**
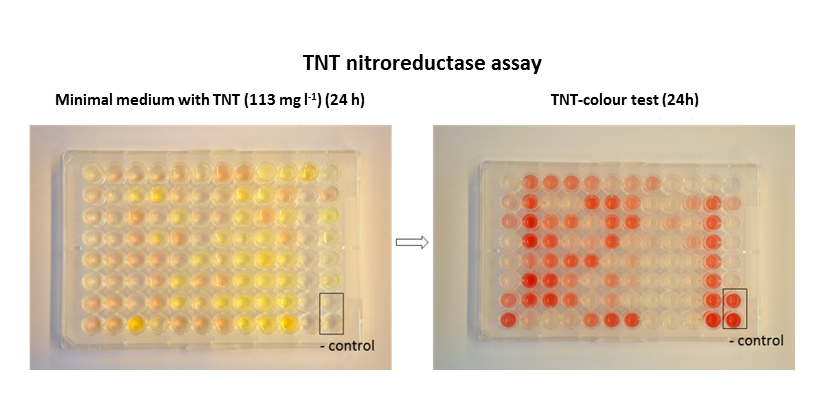
**

**Figure S2:** **TNT nitroreductase assay.** Photographs of the 96-well microplate system for the detection of TNT nitroreductase activity. Left, colour changes in the TNT-medium after 24 h produced by the bacteria. Right, microplate results after the TNT-test. Negative control was non-inoculated medium. Yellow, is amino-reduction products accumulation, orange is Meisenheimer product formation, colorless is non-inoculated medium. On the right, red is TNT, colourless is complete reduction of TNT to amino-reduction products. The medium used was a minimal salts medium supplemented with TNT (113 mg l^-1^), 53 mg l^-1^ NH_4_Cl and glucose (0.3%).

**Table S1: Origin, sequence ID, 16S identity and characteristics of isolated bacteria**

**Table S2: PGP-potential and nitroreductase activity per genus**
